# Metal Sensitization in Patients That Underwent Patch Test Pre and Post‐Implant Exposure

**DOI:** 10.1111/cod.70132

**Published:** 2026-03-09

**Authors:** Nicholas Zampa, Ilaria Lazzarato, Marcella Mauro, Alice Tassinari, Luca Cegolon, Francesca Larese Filon

**Affiliations:** ^1^ Clinical Unit of Occupational Medicine University of Trieste Trieste Italy

## Abstract

**Background:**

Sensitization to metals can cause allergic symptoms in subjects that under go device implantation but there is debate on the value of pre‐implant patch testing.

**Objectives:**

To evaluate metal sensitization in patients that underwent patch test pre and post‐implant insertion.

**Methods:**

Consecutive patients that underwent patch testing for metals before (*n*. 665) or after device implantation (*n*. 217) from January 1998 to December 2022 were investigated. Patch test were performed to investigate sensitization to Al, Au Co, Cr, Cu, Hg, Ni, Pt, Pd, Ti, Va, and Zn. Univariate and multivariable logistic regression were used to investigate factors associated to metals' sensitization.

**Results:**

A total of 882 patients were patch tested: 665 without contact dermatitis, that performed the test before device implantation and 217, with symptoms, that did the test after device implantation to verify metals sensitization for the onset of adverse reactions. In both groups, women are more represented (AdjOR 1.80; 95% CI 1.01–3.21 and 2.08; 95% CI 1.03–4.17, respectively). History of jewellery allergy significantly predicted metal sensitization, but only in the pre‐implant group (AdjOR 6.11; 95% CI 3.82–9.77). No significant differences in metal sensitization was found in the groups considered.

**Conclusions:**

Sensitization to metals were similar in pre‐implant and post‐implant patients. Women with jewellery allergy are at higher risk to have a patch test positive to metals. Post‐implant testing in case of contact dermatitis could be helpful to identify culprit allergens. Pre‐implant testing in a selected population with history of contact dermatitis or jewellery allergy could be useful to choose the right implant.

## Graphical abstract

- Sensitization to metals was similar in pre‐implant and post‐implant patients.
- Women with jewellery allergy are at higher risk to have a patch test positive to metals.
- Post‐implant testing in case of contact dermatitis could be helpful to identify culprit allergens.

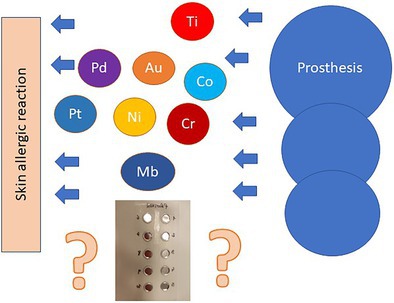

## 1 Introduction

The role of metal sensitization in skin and other reaction after implantation is debated and the need to test patients pre‐implantation is suggested only by the American Contact Dermatitis Society in subjects with a history of metal reactions [1] and in Germany Bruze suggested using titanium implants in patients with previous metal allergy [2]. Thyssen et al. in 2011 were against routine patch testing prior to surgery unless the patient has already had implant surgery with complications suspected to be allergic or has a history of clinical metal intolerance of sufficient magnitude to be of concern to the patient or a health provider [3]. UK orthopaedics in 2013 stated that metal allergy screening prior to surgery is not essential [4] and Middelton and Toms [5] in 2016 stated that there is no justification to use ‘hypoallergenic’ implants in patients with metal sensitivity for total knee replacement [4, 5]. More recently, Badell et al. [6] suggested underdoing patch tests pre‐implantation in case of foot and ankle surgery in patients with a positive history of metal allergy with the aim to choose the right device. In fact, in sensitized subjects, there is evidence of contact dermatitis and other symptoms induced by the prosthesis that improve after revision [7, 8, 9, 10, 11, 12].

To the best of our knowledge, no data are available in Italy on metal sensitization in patients before orthopaedic or dental prosthesis insertion. Moreover, in our country, sensitization to nickel is more common than other EU Countries, mainly in older women that were sensitised with metal objects before the implementation of EU Directive on nickel release from jewellery [13] and this sensitization is the principal cause of allergic contact dermatitis in the Italian population [13]. Cobalt and chromium allergy is less frequent and involved particularly workers in certain industries (metal workers, bricklayers) [14, 15], but it could be associated rarely with prosthesis reactions.

Orthopaedic and dental prosthesis can contain chromium, cobalt, nickel or other metals and can potentially induce metal‐sensitive allergies [16]. Wear and corrosion of metal implants leads to the release of free ions which can deposit in periprosthetic sites, as well as extra‐cutaneously in liver, spleen and kidneys [17, 18, 19, 20, 21]. Older orthopaedic implants were primarily composed of stainless steel alloys containing nickel, chromium, and other metals, or chromium/cobalt/molybdenum alloys [22]. These materials exhibited increased susceptibility to corrosion and mechanical wear, resulting in the release and systemic deposition of metals such as nickel, cobalt, and chromium in biological fluids and tissues, which correlated with elevated rates of metal sensitization [23, 24, 25]. In contrast, contemporary implants are designed to minimise postoperative complications and are typically fabricated from titanium‐free for cobalt and chromium, or from vitallium, a novel chromium/cobalt alloy with improved biocompatibility [25, 26, 27, 28, 29]. Additionally, individuals with active autoimmune diseases, characterised by heightened immune activity, could be at increased risk for developing metal allergies [30], however, this topic is controversial.

Up‐to‐date the practice of pre‐implant testing remains controversial [1, 4, 5], but recent studies suggested the need to patch test subjects with a history of metal allergy. In Friuli Venezia Giulia (Italy) region, pre‐implant testing was mandated by orthopaedic specialists due to the elevated prevalence of nickel sensitization observed among middle‐aged women [13]. Patch test was proposed to patients with a history of metal allergy or previous contact dermatitis [31].

The aim of this study was to determine the prevalence of metal sensitization among patients who underwent patch testing at the Allergy Clinic of the Occupational Medicine Clinical Unit between 1998 and 2022, either prior to the implantation of dental or orthopaedic prostheses or following prosthesis application in cases of suspected allergic contact dermatitis.

## 2 Patients and Methods

Patients patch tested with metals at the Allergy Center of the Occupational Medicine Clinical Unit at University of Trieste (Italy) between 1998 and 2022 were involved prior to the application of orthopaedic or dental prostheses to exclude pre‐existing sensitization (pre‐implant patients) or in case of contact dermatitis to evaluate potential sensitization following the placement of prosthetic devices (post‐implant patients).

### 2.1 Patch Tests

The patch test included cobalt (II) chloride hexahydrate 1% pet, palladium (II) chloride 1% pet, nickel (II) sulfate hexahydrate 5% pet, potassium dichromate 10% pet, zinc 2.5% pet, copper (II) sulfate pentahydrate 2% pet, copper (I) oxide 5% pet, colloidal silver 0.1%, silver nitrate 1% aq, tin 50% pet, ammonium tetrachloroplatinate (II) 0.25% aq, vanadium (III) chloride 1% pet, titanium 10% pet, gold (I) sodium thiosulfate dihydrate 0.5% pet, ammonium molybdate (VI) tetrahydrate 1% aq, aluminium (III) chloride hexahydrate 2.0% and dental amalgam. All haptens were purchased from Chemotechnique MB Diagnostics (Sweden) or FIRMA SPA (Florence).

Safety data sheets (SDS) of the materials intended for use or used in their prostheses were asked of the surgeons; consequently, metals patch tested varied according to the specific composition detailed in the SDS. In addition to the metal allergens, patients were evaluated using the European baseline series of patch tests.

Patch tests were performed on the upper back using Finn chambers on Scanpor tape. The first reading with test removal was performed at 48 h (D2), and the final reading was performed at 72/96 h (D3/D4) in accordance with the International Contact Dermatitis Research Group (ICDRG) guidelines [32]. Reactions were classified as follows: erythema (+); erythema with papules (++); erythema with papules and vesicles (+++), and doubtful or questionable reactions (+/−, ?) were considered negative.

### 2.2 Statistical Analysis

STATA software release 17 (STATA Corp, College Station, Texas) was used for statistical analysis. Continuous data were reported as mean and standard deviation, and categorical data were reported as numbers and percentages. Statistical differences between groups were calculated using the Student's *t*‐test for continuous variables and chi‐square statistics or Fisher's exact tests for categorical variables, according to numbers. Factors associated with sensitization to metals were investigated using univariate regression analysis. Variables demonstrating significance in univariate analysis were further examined through multivariate logistic regression to control for potential confounders. Statistical significance was set at *p* < 0.05.

All procedures performed in studies involving human participants were in accordance with the ethical standards of the institutional and/or national research committee and with the Helsinki Declaration. All patients signed an informed consent.

The study was approved by Comitato Etico Unico Regionale—Friuli Venezia Giulia n. 9624/2018.

## 3 Results

This study considered a total of 882 people, who underwent Patch Tests with metal series. Pre‐implant population (665 patients; 75.4%) without contact dermatitis that underwent the patch test prior to the placement of dental or orthopaedic prosthesis. Post‐implant population (217 patients; 24.6%) with contact dermatitis or other symptoms (swelling, pain, etc.) following prosthesis placement were assessed for allergic reactions to components of the existing prostheses. The majority of pre‐implant population underwent the test prior to orthopaedic prosthesis (94.7%) while the distribution was similar in post‐implant population (45.6% and 49.8% for orthopaedic and dental prosthesis, respectively) (Table 1).

**TABLE 1:** Reason to underdo patch test in pre and post implant population.

Reason for patch testing	Pre‐implant	Post‐implant	Total
	*N* (%)	*N* (%)	*N* (%)
Orthopaedic prosthesis (col %)	630 (94.7)	99 (45.6)	729 (82.6)
Intra‐uterine device (col%)	—	10 (4.6)	10 (11.3)
Dental prosthesis (col %)	35 (5.2)	108 (49.8)	143 (16.2)
Total n. (row %)	665 (75.4)	217 (24.6)	882 (100)

### 3.1 Pre‐Implant Population

Characteristics of the pre‐implant population are reported in Table 2. This group is significantly older than post‐implant population (mean age of 68.0 ± 10.4 vs. 59.5 ± 15.7 years, *p* < 0.001). Patients sensitised to metals were significantly younger (64.7 ± 12.5 vs. 69 ± 9.5 years) and women were at increased risk (OR 1.80; 95% CI 1.01; 3.21). Subjects sensitised to metals reported previous contact dermatitis with jewellery (OR 6.11; 95% CI 3.82; 9.77), in multivariable logistic regression.

**TABLE 2:** Characteristics of the pre‐implant population tested in subjects positive and negative to metals (in bold are reported significant values with *p* < 0.01).

	Pre‐implant metals+	Pre‐implant metals−	Total	Univariable odds ratio (95% CI)	Multivariable odds ratio (95% CI)
Number (%)	156 (23.46)	509 (76.54)	665 (100)		
Age (mean ± SD)	64.68 ± 12.5	69 ± 9.52	68.02 ± 10.43	**0.96 (0.95; 0.98)**	**0.97 (0.95; 0.99)**
Women *N* (%)	138 (88.46)	373 (73.28)	511 (76.84)	**2.80 (1.65; 4.74)**	**1.80 (1.01; 3.21)**
Familial allergy *N* (%)	47 (30.13)	135 (26.52)	182 (27.37)	1.19 (0.81; 1.77)	
Cutaneous allergies in family *N* (%)	16 (10.26)	41 (8.06)	57 (8.57)	1.30 (0.71; 2.40)	
Previous dermatitis *N*(%)	45 (28.85)	92 (18.07)	137 (20.60)	**1.84 (1.22; 2.78)**	1.04 (0.64; 1.69)
Allergy to jewellery *N* (%)	71 (45.51)	53 (10.41)	124 (18.65)	**7.19 (4.70; 10.99)**	**6.11 (3.82; 9.77)**
Respiratory symptoms *N* (%)	29 (21.17)	90 (18.83)	119 (19.35)	1.15 (0.73; 1.85)	
Rhinitis *N* (%)	20 (14.60)	56 (11.72)	76 (12.36)	1.28 (0.74; 2.23)	
Asthma *N* (%)	8 (5.84)	22 (4.60)	30 (4.88)	1.31 (0.57; 3.02)	
Rhinitis and asthma *N* (%)	1 (0.73)	12 (2.51)	13 (2.11)	0.30 (0.04; 2.33)	
Food allergy *N* (%)	19 (13.97)	49 (10.29)	68 (11.11)	1.42 (0.80; 2.50)	
Drugs allergy *N* (%)	19 (13.87)	111 (23.22)	130 (21.14)	0.53 (0.31; 0.90)	

*Note*: Differences were assessed using univariate and multivariable logistic regression and results are reported as OR and 95% CI.

Abbreviations: CI, confidence interval; OR, odds ratio.

### 3.2 Post‐Implant Population

The post‐implant population was constituted mainly of women (154; 71%; OR 2.08; 95% CI 1.03; 4.17) with a mean age of 59.5 ± 15.7 years (Table 3). Patients sensitised to metals were significantly younger (OR 0.97; 95% CI 0.95; 0.99). No differences were shown between subjects positive and negative to metals for atopic eczema and allergic respiratory symptoms. A history of previous contact dermatitis and allergy to jewellery was reported in higher prevalence in subjects sensitised to metals, but differences did not reach statistical significance.

**TABLE 3:** Characteristics of the post‐implant population tested in subjects positive and negative to metals (in bold are reported signific valuues with *p* < 0.01).

	Post‐implant metals+	Post‐implant metals−	Total	Univariable odds ratio (95% CI)	Multivariable odds ratio (95% CI)
Number (%)	74 (34.10)	143 (65.90)	217 (100)		
Age	55 ± 15.5	61.9 ± 15.35	59.5 ± 15.7	**0.97 (0.96; 0.99)**	**0.97 (0.95; 0.99)**
Women *N* (%)	58 (78.38)	96 (67.13)	154 (71.0)	1.77 (0.92; 3.41)	**2.08 (1.03; 4.17)**
Familial allergy *N* (%)	24 (32.43)	41 (28.67)	65 (29.95)	1.19 (0.65; 2.19)	
Cutaneous allergies in family *N* (%)	8 (10.81)	8 (5.59)	16 (7.37)	2.05 (0.74; 5.69)	
Previous dermatitis *N*(%)	20 (27.03)	31 (21.68)	51 (23.50)	1.34 (0.70; 2.56)	
Allergy to jewellery *N* (%)	9 (12.16)	8 (5.59)	17 (7.83)	2.34 (0.86; 6.33)	
Respiratory symptoms *N* (%)	18 (25.00)	35 (25.74)	53 (25.48)	0.96 (0.50; 1.86)	
Rhinitis *N* (%)	15 (20.83)	23 (17.04)	38 (18.36)	1.21 (0.58; 2.51)	
Asthma *N* (%)	1 (1.39)	7 (5.19)	8 (3.86)	0.26 (0.03; 2.21)	
Rhinitis and asthma *N* (%)	2 (2.78)	5 (3.70)	7 (3.38)	0.74 (0.14; 3.95)	
Food allergy *N* (%)	8 (11.11)	21 (15.44)	29 (13.94)	0.68 (0.29; 1.63)	
Drugs allergy *N* (%)	22 (30.56)	37 (27.21)	59 (28.37)	1.18 (0.63; 2.21)	

*Note*: Differences were assessed using univariate logistic regression and results are reported as OR and 95% CI.

Abbreviations: CI, confidence interval; OR, odds ratio.

### 3.3 Characteristics of Patients Positive to Metals

In the two populations examined, 324 patients tested positive for metals, 231 (34.7%) in the pre‐implant population, and 93 (42.9%) among post‐implant patients. Table 4 shows the results obtained examining the metals one by one. A higher prevalence of sensitization to nickel was observed in the post‐implant population (24.9%) compared to the pre‐implant population (20%), but the difference did not reach statistical significance. The second metal that had high prevalence was palladium with 7.1% and 6.2% in post and pre‐implant populations, respectively. Cobalt positivity ranked third, with a prevalence of 6.5% and 4.5% in post and pre‐implant populations, respectively. A total of 39 patients out of 879 (4.4%) tested positive for potassium dichromate: 24 (3.6%) in the pre‐implant patch test and 15 (7%) in the post‐implant patch test. For all the other metals tested, the prevalence was below 1% except for copper sulfate that resulted positive in 2.5% of the post‐implant population.

**TABLE 4:** Sensitization to metals in pre‐implant and post‐implant population.

	Pre‐implant *N*. 665	Post‐implant *N*. 217	Total *N*. 882
	Pos/tested (%)	Pos/tested (%)	Pos/tested (%)
Aluminium (III) chloride hexahydrate 2.0%	0/18	0/18	0/18
Ammonium molybdate (VI) tetrahydrate 1.0% aq	0/632	0/124	0/756
Ammonium tetrachloroplatinate (II) 0.25% aq	2/663 (0.30)	0/216	2/879 (0.23)
Cobalt (II) chloride hexahydrate 1.0% pet	30/665 (4.5)	14/217 (6.5)	44/882 (5.0)
Colloidal silver 0.1%	1/254 (0.4)	0/39	1/293 (0.3)
Copper (II) sulfate pentahydrate 2.0% pet.	0/266	1/40 (2.5)	1/306 (0.3)
Copper (I) oxide 5.0% pet.	3/398 (0.7)	2/161 (1.2)	5/559 (0.9)
Dental amalgam	0/52	1/99 (1.0)	1/151 (0.7)
Gold (I) sodium thiosulfate dihydrate 0.5% pet.	1/663 (0.15)	0/216	1/880 (0.1)
Nickel (II) sulfate hexahydrate 5.0% pet.	133/665 (20)	54/217 (24.9)	187/882 (21.2)
Palladium (II) Chloride 1.0% pet.	41/582 (6.2)	15/211 (7.1)	56/875 (6.4)
Potassium dichromate 10% pet.	24/665 (3.6)	15/271 (5.5)	39/882 (4.4)
Silver nitrate 1% aq.	0/398	0/161	0/559
Tin metal 50.0% pet.	1/632 (0.2)	1/106 (0.9)	2/738 (0.3)
Titanium (III) nitride 10% pet.	1/664 (0.15)	0/109	1/863 (0.1)
Vanadium (III) chloride 1.0% pet.	2/632 (0.3)	1/106 (0.9)	3/738 (0.4)
Zinc 2.5% pet.	2/664 (0.3)	0/211	2/875 (0.2)

The Table 5 reports symptoms and positive reactions to metals in relation to implant type. In the case of orthopaedic prosthesis, the majority of subjects had dermatitis over the prosthesis or in other sites (60.6%) and nearly half of them had a patch test positive to metals that was judged relevant in nearly 30% of cases and was related mainly to nickel, cobalt, and palladium sensitization. In one case, with symptoms not responsive to drug treatments, revision was effective.

**TABLE 5:** Implant type, symptoms, and positive patch test to metals with relevance in post‐implant patients.

Implant type and symptoms	*n* (%)	Patch test + *n* (%)	Relevant patch test *n* %
Orthopaedic (*n*. 99)			
Dermatitis (any distribution)	34 (34.3)	16 (47.1)	9 (26.5)
Dermatitis over the prosthesis	26 (26.3)	15 (57.7)	8 (30.8)
Pain	20 (20.2)	10 (50)	3 (15)
Swelling/inflammation	10 (10.1)	4 (40)	2 (20)
Loosening, implant failure	2 (2.0)	0	0
Other	7 (7.0)	4 (57.0)	2 (28.6)
Intra‐uterine device (*n* 10)			
Itching and dermatitis	10 (100)	2 (20)	2 (20)
Dental (*n* 108)			
Dermatitis, oral ulcers, lichen planus (any distribution)	33 (30.6)	15 (45.4)	10 (30.3)
Dermatitis, oral ulcers, lichen planus (near the implants, lips, and perioral)	34 (31.5)	16 (47.9)	10 (29.4)
Pain/burning	32 (29.6)	15 (46.9)	0
Swelling/inflammation	4 (3.7)	2 (50)	0
Loosening	2 (1.9)	1 (50)	0
Other	3 (2.8)	1 (33.3)	0

Ten women underwent patch test for itching and dermatitis after intra‐uterine device application; in two cases copper resulted positive and was considered relevant. Revision resulted in resolution of symptoms.

In case of dental implant, 61.2% had dermatitis or lichen planus, and nearly half of them were sensitised to metals. The sensitization was relevant in 29.9% of cases. In case of other symptoms, mainly burning of the mouth, no reactions were considered relevant. Revision was suggested in difficult cases (n. 3) after the failure of pharmacological treatment.

## 4 Discussion

Our study investigated metal sensitization in a large group of patients that underwent patch test pre or post‐implant application. In the best of our knowledge, this is the largest data base of patients tested for this reason.

### 4.1 Pre‐Implant Population

We found a female predisposition to metal hypersensitivity. Among 156 patients positive to patch test out of a total of 665, 138 were women (88.5%). This information is consistent with other studies that can be found in literature, which affirm that the prevalence of metal allergies is higher in women than in men [4, 30]. This may be attributed to women's tendency to be more commonly affected by degenerative bone and joint diseases and are therefore more often subjected to hip and knee replacement than men, but also to the fact that women are more often exposed to jewellery or other metal objects containing different types of metals. We observed that metal allergy affected younger subjects (64.68 ± 12.5 years vs. 69 ± 9.52 years in non‐sensitised subjects). This is in line with other studies in which women born in 1967–1975 are at increased risk to be sensitised to nickel [13], probably to higher exposure to nickel releasing objects during their life, while younger and older subjects had lower prevalence of nickel sensitization due to lower exposure but also to a reduced immunological reactivity in older people [4].

Our findings indicate that a history of allergy to jewellery significantly increases the risk to have a positive patch test for metals (OR 6.11; 95% CI 3.82–9.77), while a dermatitis in the past predicted sensitization only in univariable logistic regression analysis. This result is similar to those obtained by Tam et al. [7] in which the history of dermatitis from metal exposure obtained higher sensitivity in predicting metal allergy. No difference was shown between patient sensitised or non‐sensitised to metals for atopic status and respiratory allergic diseases.

The clinical relevance of preoperative testing for metal sensitization in patients considered for metal implants remains highly debated. Current evidence is inconclusive regarding whether such testing should influence the decision to use specific metallic materials [27]. Due to conflicting data on the impact of metal sensitization, authors rarely recommend routine preoperative patch testing to predict complications or early implant loosening [1, 3, 33]. Moreover, despite the high prevalence of nickel sensitization, allergic reaction to prosthesis is a rare phenomenon [34].

A large study, performed by Munch in 2015 [34], analysed the national Danish Knee Arthroplastic Register (*n*. 46.407) that was linked with the contact allergy patch test database (*n*. 27 020) found 327 patients registered in both databases. Contact allergy to nickel, cobalt and chromium resulted comparable in patients with or without revision surgery. However, in patients that underwent 2 or more episodes of revision surgery, the prevalence of chromium and cobalt allergy resulted marked higher, suggesting a potential role of metal allergy in patients with multiple revisions.

Routine screening for all patients prior to surgery is not indicated [3, 7], however, patients with a history of metal allergy may benefit from patch testing [3, 7] and positivity to metals can suggest to surgeons the use of prostheses with different metals. Nickel‐free metals currently represent the most practical approach to avoid implant‐related hypersensitivity, and the identification of predominant sensitizers such as nickel is critical to select biocompatible implant materials for allergic patients [29].

### 4.2 Post‐Implant Population

In the post‐implant population, age is a protective factor (OR 0.97; 95% CI 0.95–0.99) and women are at increased risk to be sensitised to metals (OR 2.08; 95% CI 1.03–4.17). Comparing to the pre‐implant population, this group is younger (*p* < 0.05), probably due to the increase of total hip and knee replacements over time [30] In this group, the prevalence of metal sensitization resulted higher in subjects with a positive history of jewellery allergy, but differences did not reach the statistical significance (OR 1.95; 95% CI 0.69–5.46).

Symptoms and metal sensitization results were similar to those reported by Tam et al. [7], with a relevance of metal sensitization in nearly 30% of subjects with dermatitis after the placement of orthopaedic prosthesis or those with dental implants. In case of other symptoms, only for a minority of cases was sensitization to metals considered relevant. Revisions were suggested for few cases, when symptoms did not improve with pharmacological treatment; two of ten subjects with itching and dermatitis following intra‐uterine device insertion were positive to copper, and removal was effective. Allergic reactions to copper are already reported in the literature [35, 36].

### 4.3 Characteristics of Patients Positive to Metals

Metal sensitization resulted similar in pre and post‐implant group, despite a mild higher prevalence for nickel, palladium, and cobalt sensitization in the post‐implant group that did not reach the statistical significance.

The patch test results for the pre‐implant and post‐implant groups exhibit considerable similarity; however, prevalences are higher in the post‐implant cohort. This observation aligns with the findings of Uter et al., who reported that contact allergy prevalence in patch‐tested clinical populations is generally higher than those observed in the general population, likely due to morbidity‐driven selection bias [37].

Tam et al. [7] and Furrer et al. in 2018 [38] found higher prevalence in nickel sensitization in the pre‐implant group compared to the post‐implant group (50% vs. 17.2% and 34.2% vs. 10.9%, respectively) while in our population the post‐implant population presented a higher prevalence of sensitization to nickel, although not statistical significance. Differences that could be explained by the selection of the population tested. In our case, pre‐implant subjects were with or without a history of metal allergy or previous dermatitis; therefore, patch test prevalence reflected those of the general population. Moreover, Tam reported data on patch tests with 2.5% or 5% concentrations of nickel sulfate, making it difficult to compare his results with ours [7].

Palladium sensitization ranked second with 6.2% and 7.1% in pre and post implant population: lower percentages compared to Tam et al. [7], that reported a prevalence of sensitization of 48.6% and 18.6% in pre and post‐implant population, respectively, but using a higher concentration (2%). Furrer et al. [38] reported higher prevalence of palladium sensitization (23.3%) but they tested Pd as sodium tetrachloropalladate 2%, that probably overestimated sensitization. Moreover, palladium sensitization is strongly related to nickel sensitization [39] and only a few cases are mono‐sensitised.

Cobalt sensitization ranked third with a positivity in 4.5% and 6.5% in pre and post‐implant groups, respectively. Again, our results are lower than those reported by Tam et al. [7] and Furrer et al. [38] that found 25% vs 10.5% and 15.1% vs 2.9% in pre and post‐implant group, respectively. Different percentages are related to selection of population tested and different geographical area, in fact Tam et al. [7] reported data on patch test performed in USA and Furrer et al. [38] in Switzerland.

Literature supports that nickel, cobalt, and palladium are the most common metals causing sensitization [30].

Potassium dichromate sensitization was observed in 39 out of 879 patients (4.4%), with 24 (3.6%) pre‐implant and 15 (7%) post‐implant. The percentage of sensitization to this metal was similar to that found by Tam is USA [7].

Copper oxide showed a 0.9% prevalence, with five patients testing positive: three (0.8%) pre‐implant and two (1.2%) post‐implant of intra‐uterine device. In both cases, revision was successful, and literature reports some case reports on copper allergy [35, 36].

Sensitization to other metals was very low in both pre‐implant and post‐implant groups, and their relevance was low. Dental amalgams had a 0.7% prevalence, with one patient (1% of 99 examined) testing positive post‐implant. Colloidal silver and vanadium (III) chloride had similar prevalence, 0.3%–0.4%. For vanadium (III) chloride, three patients were positive: two (0.3%) pre‐implant and one (0.9%) post‐implant. Zinc, tin, and ammonium tetrachloroplatinate (II) showed a prevalence of 0.2%–0.3%, and none was considered relevant. Titanium and gold had similar prevalence of 0.1%. No positivity was found for silver nitrate, aluminium, and ammonium molybdate. Our results are in line with those of Tam et al. [7] and Furrer et al. [38], for which no or limited relevance was found for metals other than nickel, cobalt, palladium, and chromium.

### 4.4 Strengths and Limitations

The strength of the present study lies in the comparative analysis between pre and post‐implantation patients in quite a large group of patients.

However, several limitations should be acknowledged. First, the selection of test substances was based on the chemical components listed in the safety data sheets of the prosthetic materials that were used, implanted, or planned for implantation. This approach was intentionally adopted to avoid patch testing with highly reactive substances in individuals without prior exposure. Secondly, no follow‐up data was available to know the progression of symptoms after patch testing, except for a few cases for which we suggested revision. Third, as metal allergy is quite frequent in the Trieste population, the relevance of sensitization in relation to prosthesis or implant is a matter of discussion between orthopaedic and dermatologist.

## 5 Conclusions

Allergic Contact Dermatitis related to implanted medical devices remains a contentious issue, with limited definitive guidelines available for surgeons, allergists, and dermatologists. The authors advocate that pre‐implantation evaluation via patch testing may be beneficial in selecting implant alloys for individuals with a documented history of metal or jewelry allergy.

Post‐implant evaluation in case of contact dermatitis or in case of other symptoms is justifiable once alternative etiologies of implant‐related adverse effects have been excluded. The removal or replacement of implanted metal devices containing allergenic metals may alleviate morbidity associated with metal hypersensitivity reactions. To advance understanding in this emerging field, well‐designed, large‐scale prospective cohort studies investigating metal hypersensitivity in implanted devices are warranted.

## Author Contributions

**Nicholas Zampa:** writing – original draft, writing – review and editing, investigation, resources, visualization, formal analysis, funding acquisition. **Ilaria Lazzarato:** data collection. **Marcella Mauro:** data collection, investigation, review and editing. **Alice Tassinari:** data collection and review and editing. **Luca Cegolon:** formal analysis, software. **Francesca Larese Filon:** study design, review and editing, data analysis.

## Conflicts of Interest

The authors declare no conflicts of interest.

## Data Availability

The data that support the findings of this study are available from the corresponding author upon reasonable request.
